# Stereolithography 3D Printing of Stimuli-Responsive Spin Crossover@Polymer Nanocomposites with Optimized Actuating Properties

**DOI:** 10.3390/nano14151243

**Published:** 2024-07-24

**Authors:** Onkar Kulkarni, Alejandro Enriquez-Cabrera, Xinyu Yang, Julie Foncy, Liviu Nicu, Gábor Molnár, Lionel Salmon

**Affiliations:** 1 Laboratoire de Chimie de Coordination (LCC), Centre National de la Recherche Scientifique (CNRS), University of Toulouse, 205 Route de Narbonne, 31077 Toulouse, France; 2 Laboratoire d’Analyse et d’Architecture des Systèmes (LAAS), Centre National de la Recherche Scientifique (CNRS), University of Toulouse, 7 Avenue du Colonel Roche, 31400 Toulouse, France

**Keywords:** stereolithography, nanocomposites, spin crossover, mechanical properties

## Abstract

We used stereolithography to print polymer nanocomposite samples of stimuli-responsive spin crossover materials in the commercial photo-curable printing resins DS3000 and PEGDA-250. The thermomechanical analysis of the SLA-printed objects revealed not only the expected reinforcement of the polymer resins by the introduction of the stiffer SCO particles, but also a significant mechanical damping, as well as a sizeable linear strain around the spin transition temperatures. For the highest accessible loads (ca. 13–15 vol.%) we measured transformation strains in the range of 1.2–1.5%, giving rise to peaks in the coefficient of thermal expansion as high as 10^−3^ °C^−1^, which was exploited in 3D printed bilayer actuators to produce bending movement. The results pave the way for integrating these advanced stimuli-responsive composites into mechanical actuators and 4D printing applications.

## 1. Introduction

The spin crossover (SCO) phenomenon is observed in certain octahedral metal complexes, where the spin state of the complex changes between the high-spin (HS) and low-spin (LS) electronic configurations due to external stimuli, such as temperature, pressure or light irradiation [1]. Spin crossover is observable in different first-row transition metal ions with 3d^4^ to 3d^7^ electron configurations, but materials that display SCO behavior most frequently comprise complexes of iron(II) [2].

Building upon Olivier Kahn’s concepts from the 1990s [3,4], the research focus on SCO materials has evolved in the past two decades progressively, towards exploring the technological applications of these stimuli-responsive molecular materials, showcasing notable alterations in magnetic, optical, electrical, thermal, and mechanical properties [5]. The present work relates mostly to potential applications in mechanical actuators. The key underlying property in this context is the significant volume change that accompanies the SCO phenomenon, which typically ranges from 1 to 10% (up to 22%) [6]. This property, which allows one to transform thermal energy into mechanical work, has been known in the SCO field for several decades, but received significant attention only recently for practical applications [7]. Since then, several proof-of-concept SCO actuation devices have been constructed, which comprised, in most cases, bilayer cantilever structures producing bending motion [8,9,10,11,12,13,14,15,16]. Notably, microelectromechanical devices were fabricated by evaporating [9,13], solvent casting [11] or spray-coating [10] sub-micrometer thick films of SCO compounds onto silicon micro-cantilevers, demonstrating not only static deflection, but also a modulation of dynamical properties (resonance frequency and quality factor) when changing the spin state of the molecules. At a more macroscopic level, bilayer SCO composite cantilevers were produced by solvent casting methods [7], which were then rendered more functional using integrated heaters [15], strain sensors [12], control systems [14] and pre-programmed movements [16].

However, to implement SCO compounds into devices it is necessary to develop more powerful processing methods, which is the central objective of the work described in this paper. Processing SCO materials in their bulk form is often not straightforward, and incorporating SCO complexes into polymer matrices appears thus a highly effective and versatile method for diverse applications [17]. In this context, the fabrication of SCO composites using computer-aided additive manufacturing is a promising area of research with potential applications in electrothermal–mechanical actuators and functional switchable micro- and macro-scale structures, often coined by the term 4D printing [18,19,20,21]. By controlling the nature of the polymer and the concentration of the SCO molecular material, optimized polymeric composites can be developed, enabling the creation of a broad range of objects, from thin films to complex and intricate structures. With this idea in mind, a few years ago, we developed a stereolithographic (SLA) 3D printing process that was used to fabricate various objects from the SCO complex [Fe(NH_2_trz)_3_]SO_4_ (NH_2_trz = 4-NH_2_-1,2,4-triazole) embedded in a commercial photo-curable printing resin [22]. The SLA apparatus uses light to crosslink and solidify a liquid monomer ink via photopolymerization. SLA allows for both high accuracy and high resolution (ca. 10 μm) of the layer-by-layer printed 3D objects, including composite materials [23,24]. This approach allowed for the preservation of the thermomechanical properties of the polymer and the stimuli-responsive properties of the SCO filler, offering a new, powerful method to process SCO materials [22]. Bimorph bending cantilevers, consisting of SCO-doped and pure resin layers, were also printed, demonstrating effective 4D printing capability. In the continuity of this previous work, we present here a more detailed investigation of this additive manufacturing method, using different photo-curable printing resins and SCO complexes at varying volume fractions, aiming at exploring the scope and limitations of this approach for the 3D printing of composite SCO materials with respect to their mechanical actuating properties.

## 2. Experimental

**Figure 1:**
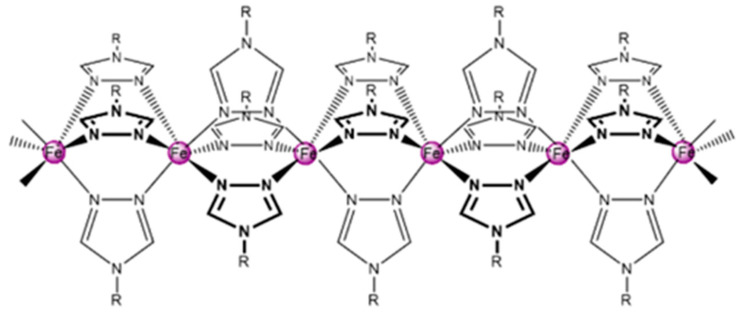
Chain-like structure of the Fe(II)-triazole family of complexes [Fe(Rtrz)_3_]_n_^2n+^ (Rtrz = 4-R-1,2,4-triazole).

We conducted experiments with two different SCO complexes, [Fe(NH_2_trz)_3_]SO_4_ (**1**) and [Fe(NH_2_trz)_3_](BF_4_)(SiF_6_)_0.5_ (**2**), belonging to the well-known family of Fe(II)-triazole complexes (Figure 1). The selected complexes were synthesized as nanocrystalline powders using well-established literature recipes [25,26]. To examine the purity, spin-crossover properties and particle morphology of the powder samples **1** and **2**, calorimetry, IR spectroscopy, X-ray diffraction and transmission electron microscopy (TEM) techniques were used (see Table 1 and Figure S1 in the Electronic Supplementary Information, ESI). Briefly, complex **1** consists of ca. 1 μm long rod-shaped particles with a form factor of ca. 10, whereas the powder of complex **2** is composed of ‘arbitrary’ shaped sub-micrometric particles with a broad size distribution. Both compounds display spin transition above room temperature and a large unit-cell volume expansion at the SCO, rendering them particularly interesting for actuator device development (see Table 1). It may be worth noting that some other practical considerations have significantly influenced the selection of the SCO complexes. Given the substantial material requirements for composite production by SLA (approximately 5 g of pure SCO solid per experiment), it is necessary to use compounds that can be efficiently and reproducibly synthesized, allowing for easy scalability. Furthermore, the Fe(II)-triazole family of compounds display only weak, Laporte-forbidden absorption bands in the visible spectral range (around ca. 540 nm), and therefore do not considerably attenuate the 405 nm laser light used in the SLA process (vide infra). Furthermore, it is of utmost importance to opt for a complex capable of forming small particles, as this property will facilitate the proper dispersion of the complex within the matrix, ensuring homogeneous distribution and optimal integration.

**Figure 2:**
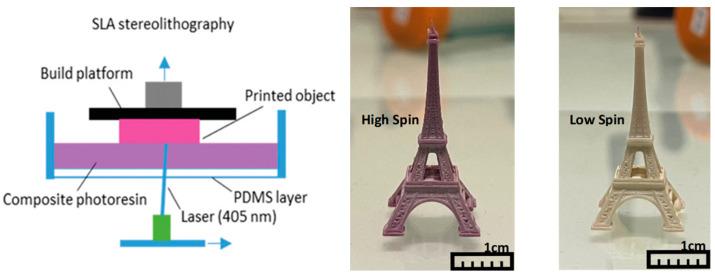
Scheme of stereo-lithography printing and photographs of a high-resolution structure made of the composite **1**@DS3000 (20 wt.%) recorded in the two spin states.

For SLA printing, we used a 29J+ printer from DWS (Thiene, Italy), incorporating galvanometric mirrors for laser beam movement (Figure 2). The laser (405 nm, 50 mW), with a diameter of 20 μm, traverses the *x* and *y* directions at a speed of 500 μm/s. The *z*-axis moving stage, supporting the sample holder and tank, ensures a minimum layer thickness of 10 μm, but in practice, we used resolutions between 20 and 50 μm, depending on the size of the printed objects. Notably, for the mechanical tests, rectangular beams of 30 (*x*) × 8 (*y*) × 0.2 (*z*) mm^3^ were printed with 20 (*z*) − 30 (*x*,*y*) μm resolution. To prevent adhesion of the printed object to the vat’s surface, poly(dimethylsiloxane) covers the vat bottom, inhibiting resin cross-linking. We explored the use of two different resins, DWS DS3000, an acrylic-based proprietary photo-curable printing resin, and PEGDA-250 (polyethylene glycol diacrylate, Sigma Aldrich, Saint-Louis, MO, USA). Unlike DS3000, which is pre-formulated, derivatives of polyethylene glycol require adding a photoinitiator, which was phenylbis(2,4,6-trimethylbenzoyl) phosphine oxide (Irgacure 819, Sigma Aldrich) in our case, at a concentration of 0.1 wt.%. These photoinitiators absorb light energy and generate reactive species, such as free radicals or cations, which initiate the crosslinking or polymerization reactions in the resin. To obtain a homogeneous dispersion of the particles in the composites, the SCO powder samples used for the printing were mixed with the monomer solutions (up to 38 wt.%) using a handheld mechanical homogenizer for approximately 20 min.

Scanning electron microscopy (SEM) pictures of the SLA-printed composites and TEM images of the SCO particles were recorded using a Hitachi (Tokyo, Japan) S-4000 and a JEOL (Tokyo, Japan) JEM-1011 instrument, respectively. TEM samples were prepared by placing a drop of the particles (suspended in ethanol) on a carbon-coated copper grid, whereas specimens for SEM were obtained by breaking the composite (cooled by liquid nitrogen) and metallizing the cross-section with Pt. Energy-dispersive X-ray spectroscopy (EDS) analysis, coupled with SEM, was conducted using a JEOL JSM 6700F—EDS instrument.

Differential scanning calorimetry (DSC) measurements were conducted using a NETZSCH (Selb, Germany) DSC 3500 Sirius equipment at scan rates of 10 °C/min under nitrogen gas purge (40 mL/min). Temperature and heat flow were calibrated using the melting transition of pure indium metal. The typical uncertainty regarding the heat of transformation is estimated as 10%, due to the subtraction of the unknown baseline. Viscosity measurements were carried out using an Anton Paar (Graz, Austria) MCR302 rheometer.

Dynamic mechanical analysis (DMA) were carried out using a DMA850 instrument (TA Instruments, New Castle, DE, USA), providing resolutions of force to 10^−5^ N, displacement to 0.1 nm, and phase lag to 10^−5^ deg. DMA measurements were conducted at a temperature scan rate of 3 °C/min using an initial static force of 0.1 N, which was continuously adjusted, proportional (150%) to the sample stiffness variation, to ensure the dynamical force remained inferior to the static force all through the experiments. From the DMA measurements, we assessed the static elongation ($\Delta l_{s}$) and the dynamical elongation modulus (E*) as a function of the temperature. The modulus was measured in displacement control mode with an imposed sinusoidal strain of 1 Hz in frequency and amplitude of 12 µm. In our uniaxial tensile experimental configuration, E* is given by

(1) $$E^{*}\left(T\right)=E^{\prime }\left(T\right)+iE^{\prime \prime }\left(T\right)=\frac{F_{D}}{{\Delta l}_{D}}\left(\frac{l}{wh}\right)cos\delta +i\frac{F_{D}}{{\Delta l}_{D}}\left(\frac{l}{wh}\right)sin\delta$$

Here, ${\Delta l}_{D}$ represents the amplitude of dynamic elongation, $F_{D}$ is the applied dynamic force amplitude, *δ* signifies the phase difference between imposed stress and resulting strain, and *l*, *w*, and *h* denote the initial length, width, and thickness of the composite film, respectively. The real part of the complex modulus is denoted as the storage modulus (*E*′), while the imaginary part (*E*″) is referred to as the loss modulus. *E*′ is directly proportional to the maximum elasticity stored in the material during each cycle, reflecting the elastic component in the material’s viscoelastic behavior and characterizing its stiffness. On the other hand, *E*″ is proportional to the energy dissipated as heat in each cycle, reflecting the viscous aspect of the material’s viscoelastic behavior and indicating the material’s damping. Damping, in this context, is the energy dissipation by a material under cyclic loading and is quantified as the tangent of the phase angle *δ*, which is a dimensionless number, calculated as the ratio of the loss modulus to the storage modulus (tan *δ* = *E*″/*E*′).

## 3. Results and Discussion

**Figure 3:**
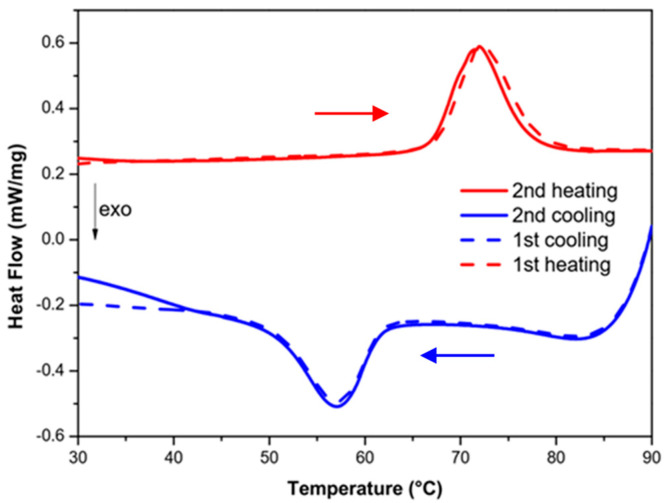
DSC thermogram of an [Fe(NH_2_trz)_3_]SO_4_@DS3000 composite sample (30 wt.%) acquired for two successive heating (red) and cooling (blue) cycles. (Arrows indicate the direction of temperature change).

For thermal and mechanical characterizations, simple stripe-like composite structures were printed. In addition, to demonstrate the potential of our SLA approach, we also printed more complex composite objects with the highest resolution (ca. 10–20 μm) allowed by our printer (Figure 2). In each case, the SCO properties were easily inferred from the color change by heating (cooling) the printed materials above (below) their spin-transition temperatures. This thermochromism can be attributed to metal-centered absorption bands, which appear in the visible spectral range (near 540 nm) in the LS state and in the near-infrared spectral range (around 800 nm) in the HS state [1]. Representative SEM images of the SLA printed neat polymer and composite samples are shown in Figures S2 and S3 in the ESI. One can clearly observe the separation of the successively printed layers in the PEGDA-based samples, whereas this effect is much less pronounced in the DS3000 resin. The particle dispersion is fairly homogeneous in both polymers, though some aggregates appear in both cases. The SCO characteristics of composites can be evaluated in a quantitative manner through DSC measurements. As a typical example, Figure 3 shows the DSC thermogram for a **1**@DS3000 (30 wt.%) composite (see Figure S4 for the other DSC data). It reveals an endothermic peak on heating near 72 °C and an exothermic peak near 56 °C on cooling, associated with the SCO phenomenon—similar to the pure powder of 1 (see Figure S1). No other thermal event attributable to the polymer matrix is apparent in the investigated temperature range. One can remark that the endothermic peak on the first heating is slightly shifted to higher temperatures. This so-called run-in effect is typical for all samples studied and originates from different reasons, including solvent loss, microstructural changes, stress release and so forth. Following the first thermal cycle, the successive transitions were well reproducible both for heating and cooling, for each sample.

Table 2 summarizes the spin-transition temperatures and the calorimetric data obtained for the different SLA printed composite samples. Interestingly, we observed systematically a small reduction in the hysteresis width in the composites in comparison to the neat powder (see Table 1), in particular on the cooling branch. This finding indicates possible changes in the nucleation energy barrier associated with the HS to LS phase transition. Yet, the overall behavior of the particles embedded in the SLA resins remains comparable with that of the bulk sample. Since the heat of transformation associated with the SCO phenomenon is proportional to the particle weight fraction, using the calorimetric data in Table 2 we could assess the effective concentration (per weight and per volume) of the particles in the different 3D printed composites. In each case, we have found a somewhat reduced effective concentration with respect to the targeted one. This observation indicates a slight sedimentation of particles from the composite resin suspensions during the relatively long printing process. It is important to stress that the presence of SCO particles can significantly interfere with the SLA process via different mechanisms. First, the agglomeration of particles is a common challenge that can affect the uniformity of the printed parts and the overall printing quality. Additionally, high particle concentrations lead to increased viscosity, potentially hindering the flow, and thus the printing, of subsequent layers. Moreover, the light-penetration depth and thus the cure time are also affected by the presence of SCO particles in the resin. Overall, taking into account these limitations, as well as the decrease in the particle concentration due to the relatively long processing times, we were able to print composites with particle concentrations up to ca. 19 vol.% for the PEGDA matrix, whereas for the DS3000 resin the particle volume fractions were limited to ca. 13 vol.%. In fact, for 30 wt.% SCO particles, the viscosity of the DS3000 composite resin (69 Pa·s) is higher than that of the PEGDA composite resin (12 Pa·s). All the viscosity measurements are gathered in Table 2.

**Figure 4:**
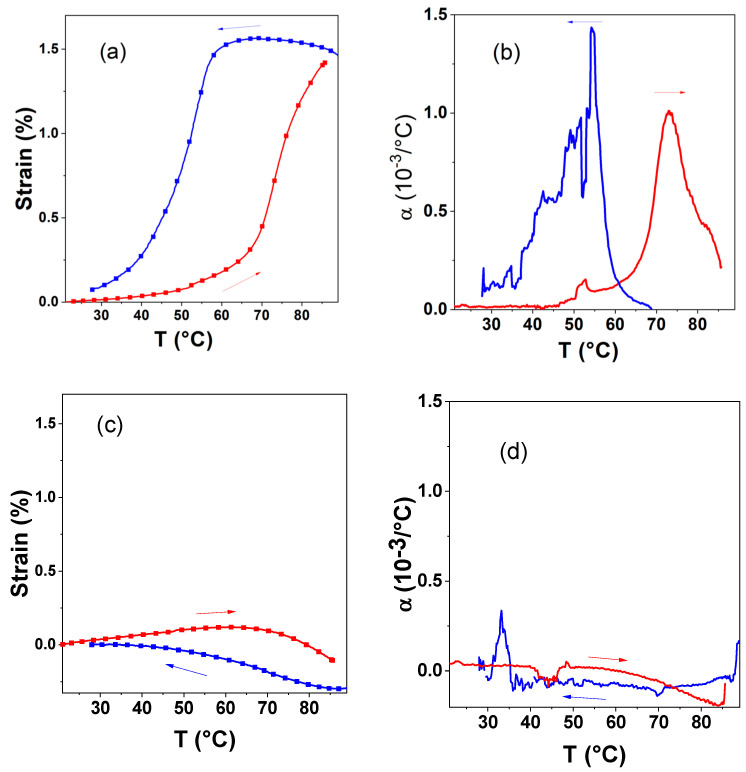
(**a**) Thermal strain and (**b**) coefficient of thermal expansion in an SLA printed **1**@DS3000_30 composite sample (ca. 13 vol.%) as a function of the temperature. Heating (red) and cooling (blue) refer to the second thermal cycle. For comparison, the same experiments are also shown for the neat DS3000 resin in (**c**) and (**d**). (Arrows indicate the direction of temperature change).

**Figure 5:**
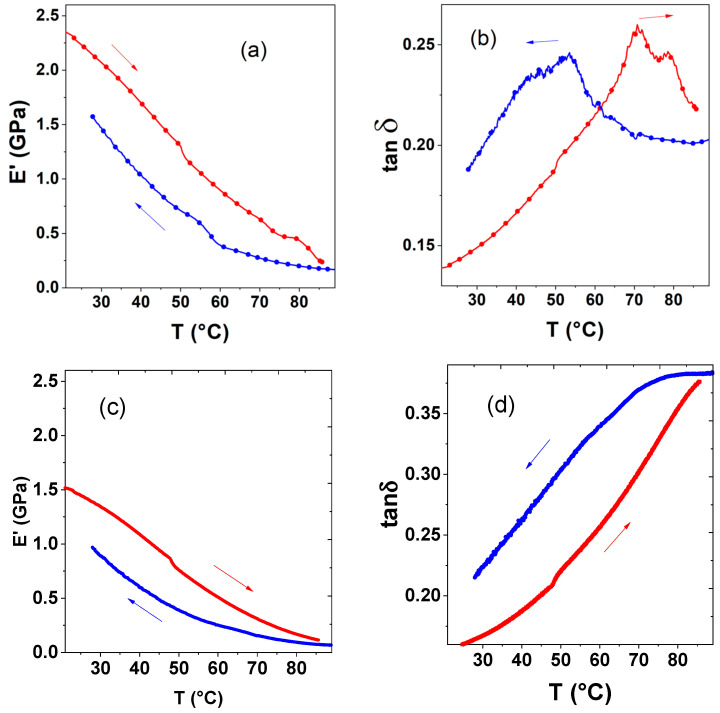
(**a**) Storage modulus and (**b**) loss tangent in an SLA printed **1**@DS3000_30 composite sample (ca. 13 vol.%) as a function of the temperature. Heating (red) and cooling (blue) refer to the second thermal cycle. For comparison, the same experiments are also shown for the neat DS3000 resin in (**c**) and (**d**). (Arrows indicate the direction of temperature change).

In order to evaluate the thermomechanical properties of the SLA printed composites, DMA measurements were carried out on several specimens for each composite, as well as for the neat polymers, printed using similar parameters (see Figure 4 and Figure 5 for the composite **1**@DS3000_30 and the neat DS3000 resin, whereas DMA data for the other samples are shown in Figures S5–S11 in the ESI). The main thermomechanical properties extracted from the DMA measurements are tabulated in Table 3 for each sample.

The storage modulus of the SLA printed pure DS3000 specimens was approximately 1.5 GPa at room temperature, whereas its loss modulus was ca. 240 MPa, denoting a loss tangent of ca. 0.16. Upon heating to 90 °C, the storage and loss moduli show a monotonous softening down to ca. 80 and 30 MPa, respectively (see Figure 5), giving rise to an overall increase of tan*δ* to a value of 0.38. As it can be expected, in parallel, the sample length expands steadily with an associated linear thermal-expansion coefficient of ca. 10^−5^ °C^−1^, followed, however, by an unusual decrease above ca. 60 °C. The latter observation indicates the occurrence of a (reversible) thermally induced process in the polymer resin, whose nature remains to be elucidated. As shown in Figure S5 in the ESI, both the room-temperature storage modulus (800 MPa) and loss modulus (140 MPa) of the neat PEGDA-250 sample are lower than that of the DS3000 resin, but the resulting loss tangent (0.17) is similar. At first sight, the temperature dependence of *E*′ and *E*″ are comparable between the two polymer matrices, with a significant softening for increasing temperatures in both cases. However, the thermal behavior of the loss tangent reveals a significant difference, with a marked loss peak near 40 °C in the PEGDA sample (Figure S5). As a result of this damping process, the thermal expansion behavior of this sample is non-monotonous (Figure S5) with a quasi-zero expansion below ca. 50 °C and a linear expansion at a rate of ca. 10^−4^ °C^−1^ above this temperature.

As summarized in Table 3, quite expectedly, the addition of stiff SCO particles into the softer polymer matrix leads to increase in the storage modulus (*E*′). Yet, it is worth noting that besides the particle volume fraction, other parameters also play a role in establishing the effective mechanical properties. The primary factors influencing the mechanical properties are the presence of porosity, the crosslinking fraction of monomer within the specimens, and the particle distribution. It was not possible to separately analyze these parameters, but obviously the increasing particle load gives rise to reduced photo-curing efficiency, as well as to an increasing probability of particle–particle interactions (through aggregation), both effects potentially leading to reduced mechanical properties.

When switching from the low-spin to the high-spin state, the most apparent mechanical effect is a distinct elongation of the sample at the spin transition (Figure 4a). This strain proves reversible, as it is mirrored by an equivalent contraction during the cooling phase while the sample returns to the LS state. (Note that the spin transition temperatures measured in DMA and DSC differ due to the different thermal lags involved in these techniques). The observation of this SCO-induced strain in the composite signifies the effective transmission of the mechanical strain associated with the SCO in the particles into the polymer resins. In a first crude approximation, taking a simple rule of mixtures, we can hypothesize that the strain arising from the SCO phenomenon (*ε_SCO_*) in the composites is proportional to the volumetric expansion of the particles at the SCO (Δ*V_SCO_*/*V*), and the proportionality factor equals one-third of the particle volume fraction (*f*):

(2) $${\epsilon_{SCO}}^{composite}={{f\epsilon }_{SCO}}^{particle}=f\frac{{\Delta V}_{SCO}}{3V}$$

The factor 1/3 comes from the assumption of a random particle orientation in the matrix (i.e., isotropic composite). To test this hypothesis, we conducted experiments with DMA test samples obtained from the same composite resin suspension using different printing directions (*x*, *y*, *z*), and, indeed, we could not observe a sizeable difference of *ε_SCO_* among these samples. The bulk [Fe(NH_2_trz)_3_]SO_4_ sample displays a volume strain of ca. 5% (Table 1). By examining the data in Table 3, we can thus deduce that the SCO-induced macroscopic strain in the composites systematically surpasses the prediction of Equation (2). Notably, for the sample **1**@DS3000_30, which exhibits the highest strain (1.5%), the predicted value is only 0.22%. This difference between the experiment and Equation (2) cannot be accounted for by a deviation from random particle orientation. Indeed, since the entire transformation strain is developed in the crystallographic *c*-direction, even with the assumption of a perfect alignment of each particle with respect to the direction of the DMA measurement, which is obviously not the case here, the proportionality factor would be multiplied only by three, still far from the experimental results. To understand this discrepancy, we should refer to the work of McMeeking [28], as well as to Rosen and Hashin [29], who obtained an exact formula for the effective transformation strain in two-phase composites as

(3) $${\epsilon_{SCO}}_{ij}^{composite}={{f\epsilon }_{SCO}}_{ij}^{particle}+C{\epsilon_{SCO}}_{kl}^{particle}$$

where C is defined by

(4) $$C=P_{klmn}\{S_{mnij}^{composite}-{[fS}_{mnij}^{particle}{+\left(1-f\right)S}_{mnij}^{matrix}]\}$$

and $P_{klmn}$ is defined by

(5) $$P_{klmn}{(S}_{mnij}^{particle}{-S}_{mnij}^{matrix})=I_{klij}$$

with *S* being the elastic compliance tensor and *I* the identity tensor. Equation (3) highlights clearly the difference between the actual transformation strain in the composite and the rule of mixtures given by Equation (2). The origin of this difference comes from the fact that the effective elastic compliance of the composite is not a simple volume average of the compliances of the constituents, for which reason C in Equation (4) is non-zero. In fact, using Equations (3)–(5), it has been shown previously [30] that embedding SCO particles in a soft (resp. stiff) matrix, results in an increase (resp. decrease) in the effective strain with respect to the expectation of Equation (2). Indeed, the latter is only valid when the elastic properties of the particles and the matrix are similar.

As shown in Figures S9 and S10, despite the somewhat different elastic properties of the PEGDA matrices in comparison with the DS3000, both polymers afford a similar actuating performance for similar particle loads. The notable advantage of PEGDA in the present context resides in the fact that, using this resin, we were able to reach particle loads near 15 vol.% more consistently, in comparison with the DS3000 resin. One must note, however, that for the samples with the highest nominal particle loads (**1**@PEGDA_38 and **1**@DS3000_35) the transformation strain starts to become reduced, which indicates the limits of the useful charge, wherein adverse effects (e.g., particle aggregation) are gaining importance. Finally, for composites with particles of the SCO complex **2**, a similar expansion/contraction is observed at the SCO (Figure S11), but at distinct transition temperatures, demonstrating the possibility of adjusting the actuation temperatures by chemical modification of the composition of the particles.

If we differentiate the strain (*ε*)-vs.-temperature curves, we can obtain the coefficient of linear thermal expansion (*α*), which is defined as the fractional change in length per degree of temperature change:

*α* = *dε*/*dT* (6)

Far from the spin-transition temperatures, the thermal expansion has low values, comparable with that observed for the pure polymers. However, around the spin transition, *α* reaches peak values of ca. 10^−3^ °C^−1^ (Figure 4b). This ‘colossal’ thermal expansion, which exceeds by two orders of magnitude the expansion of the pure polymer, is the key property of interest for mechanical actuating applications.

As shown in Figure 5a (see also the ESI), the storage modulus (*E*′) of the composites appears to be not much affected by the spin transition, albeit there is a small change in slope in the *E*′-vs.-*T* curves near the SCO temperature range. Nevertheless, the mechanical interaction between the particles and the matrix becomes evident when looking into the loss-tangent behavior, characterized by prominent dissipation peaks (tan *δ* = 0.25) around the spin-transition temperatures (Figure 5b, see also the ESI). As discussed in Ref. [30], this phenomenon can be accounted for by the sensibility of the spin-state equilibrium to mechanical stress. Because the relaxation of the spin state of the particles upon an applied stress gives rise to some strain, it facilitates the deformation; i.e., the elastic modulus appears reduced near the spin-transition temperature. In other words, the spin-state of the particles provides an ‘anelastic’ link between stress and strain, besides the usual elastic response. Because the relaxation process is thermodynamically irreversible, energy will be dissipated, which is manifested by the mechanical damping peaks of tan *δ*.

**Figure 6:**
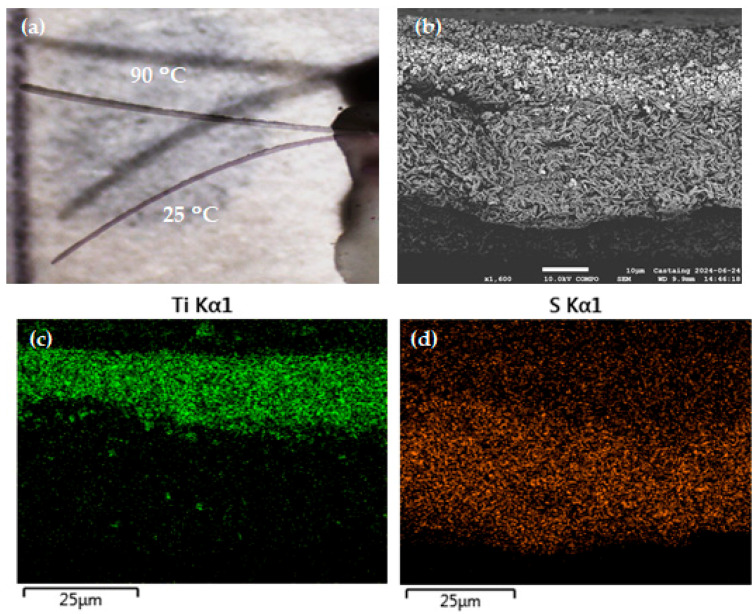
(**a**) Overlaid photographs showing the bending movement of a rectangular bilayer cantilever (**1**@PEGDA/BTO@PEGDA) upon the spin transition when heating from 25 to 90 °C. (**b**) Compositional backscattered electron SEM image of the cross-section of the bilayer. EDS elemental maps of the bilayer cross-section for (**c**) titanium and (**d**) sulfur.

Finally, in order to demonstrate the actuating ability of the SLA printed composite materials, we have printed a bilayer beam structure of 5 mm length, 0.5 mm width and 0.045 mm thickness. The active layer was printed using the **1**@PEGDA_30 slurry, whereas the inactive layer consists of PEGDA-250 loaded with 66 wt.% of BaTiO_3_ particles of mean size ca. 600 nm. The aim of using these oxide particles in the present case is twofold: it allows us to reinforce the structure, and it also affords a reduced thermal expansion coefficient (ca. 10^−5^ 1/°C) with respect to the neat PEGDA-250. As such, the mismatch of thermal expansion between the two strata is enhanced, affording more ample thermally induced bending movements. Chemical composition-sensitive SEM imaging, coupled with EDS elemental maps, reveals a clear separation of the two printed layers (Figure 6b–d). Meanwhile, the common polymer matrix between the two layers ensures their good adhesion, avoiding delamination problems upon the bending movement. Indeed, as shown in Figure 6a, in a single-clamp configuration, when heated in an oil bath, the free end of the actuator exhibits a large, reversible tip displacement of ca. 3 mm upon thermal cycling between the low-spin and high-spin states of the SCO complex. Using the classical Timoshenko beam theory (see Ref. [7] for the relevant equations), this allows us to estimate the reactive force at the end of the cantilever as ca. 0.15 mN.

## 4. Conclusions

The unique characteristics of spin-crossover complexes, such as the sudden, reversible expansion at the low-spin to high-spin transition and the associated large work density, make them a compelling choice for mechanical actuation applications in various domains such as micro- and nanoelectromechanical systems, soft robotics, and biomedical devices [8]. In this context, the use of emerging additive manufacturing technologies offers the potential for fabricating 3D structures with complex and accurate geometries, making these methods highly appealing for the development of advanced composite SCO actuators. With this idea in mind, in the present work we explored the use of stereolithography for printing SCO@polymer composites, aimed for mechanical actuation purposes. It turns out that the application of SLA in the fabrication of composites incorporating SCO particles as fillers presents certain limitations, primarily due to the increasing viscosity of the composite resin suspensions. Indeed, SLA requires a slurry with relatively low viscosity to ensure unhindered flow under the printing head, thereby facilitating successful printing of the successive layers. Another challenge is the necessity to achieve homogeneous dispersions of the SCO particles within the composite matrix, which is of utmost importance for the desired actuating properties. Despite these challenges, we have clearly confirmed the potential of the SLA method for producing custom-tailored SCO-polymer composite objects. We have also demonstrated the versatility of this approach by using different photo-curable printing resins in combination with different SCO complexes, displaying distinct mechanical and spin-transition properties. Calorimetry analysis has shown that the SCO properties of the particles were preserved in the 3D printed objects, albeit with a small reduction in the targeted particle load, which we have attributed to losses due to particle sedimentation. The thermomechanical analysis of the SLA-printed objects revealed not only the expected reinforcement of the polymer resins by the introduction of the stiffer SCO particles, but also a significant mechanical damping around the spin-transition temperatures, which we have ascribed to anelastic effects conveyed by the SCO particles. Most importantly, these composite samples exhibit a sizeable linear strain at the spin transition. For the highest possible particle loads (ca. 13 vol.% in DS3000 and 15 vol.% in PEGDA-250) we measured transformation strains in the range of 1.2–1.5%, giving rise to peaks in the coefficient of thermal expansion as high as 10^−3^ °C^−1^. These values are in excess with respect to the neat ‘particle transformation strain’, which we attributed to the amplification effect of the soft polymer matrices. Finally, to demonstrate the actuating capability of the 3D printed SCO composite materials, a bilayer actuator was fabricated and tested, showing considerable bending movement and associated reactive force. Work is currently under way aiming for the optimization of the rheological properties of the composite resin suspensions, which could help to optimize the effective mechanical properties of the printed composite actuators. We envision also printing multi-material devices combining the actuating properties of SCO compounds with piezoelectric composites, which could serve for thermo-electromechanical transduction purposes.

## Figures and Tables

**Table 1:** SCO complexes used in this work and their key properties: volume strain associated with the SCO (Δ*V_SCO_*/*V*), spin-transition temperatures (*T*_1/2_↑ on heating and *T*_1/2_↓ on cooling), enthalpy change at the spin transition (Δ*H*), molar mass (*M*) and mass density (*ρ*) at room temperature.

SCO Complexes	Δ*V_SCO_*/*V* (%)	*T*_1/2_↑ (°C)	*T*_1/2_↓ (°C)	Δ*H* (J/g)	*M* (g/mol)	*ρ* (g/cm^3^)	Ref.
**1**: [Fe(NH_2_trz)_3_]SO_4_	5	71	53	48	407.1	2.086	[27]
**2**: [Fe(NH_2_trz)_3_](BF_4_)(SiF_6_)_0.5_	7	46	36	51	476.7	1.992	[26]

**Table 2:** Spin-transition temperatures and associated enthalpy changes in the different SLA printed composite samples inferred from DSC measurements. Both the targeted and effective filler concentrations are noted (The mass densities of DS3000 and PEGDA-250 are 1.0 and 1.1 g/cm^3^, respectively). The viscosity, *η*, of the composite resins used for printing is also noted.

Sample Name	Target wt.%	*T*_1/2_↑ (°C)	*T*_1/2_↓ (°C)	ΔH (J/g)	*η* (Pa·s)	Effective wt.%	Effective vol.%
**1**@DS3000_15	15	71	53	3.7	9.5	8	4
**1**@DS3000_20	20	71	56	6.2	19	13	6.5
**1**@DS3000_30	30	72	56	12.3	69	26	13
**1**@DS3000_35	35	72	53	10.8	140	23	11.5
**1**@PEGDA_30	30	70	55	13.5	12	28	15
**1**@PEGDA_38	38	76	57	17	---	35	19
**2**@PEGDA_30	30	51	41	10.7	---	21	12

**Table 3:** DMA data obtained for the neat DS3000 and SCO@DS3000 composite samples.

Sample Name	vol.%	*E*′ (MPa) @ 20 °C	*E*″ (MPa) @ 20 °C	SCO Strain (%)
Neat DS3000	0	1500	240	N/A
**1**@DS3000_15	4	1900	250	0.2
**1**@DS3000_20	6.5	1800	220	0.3
**1**@DS3000_30	13	2300	290	1.5
**1**@DS3000_35	11.5	4100	290	0.6
Neat PEGDA	0	800	140	N/A
**1**@PEGDA_30	15	1300	230	1.2
**1**@PEGDA_38	19	1500	180	0.9
**2**@PEGDA_30	12	2200	150	0.4

## Data Availability

The raw data supporting the conclusions of this article will be made available by the authors on reasonable request.

## Supplementary Materials

The following supporting information can be downloaded at: https://www.mdpi.com/article/10.3390/nano14151243/s1, Figure S1: DSC thermograms, FTIR spectra, powder XRD patterns (Cu K-alpha) and representa-tive TEM images for the powder samples of [Fe(NH_2_trz)_3_]SO_4_ (left panel) and [Fe(NH_2_trz)_3_](BF_4_)(SiF_6_)_0.5_ (right panel). Figure S2: Representative SEM images of the neat SLA printed PEGDA film (left column) and the **1**@PEGDA (30 wt.%) composite sample (right column). Figure S3: Representative SEM images of the neat SLA printed DS3000 film (left column) and the **1**@DS3000 (30 wt.%) composite sample (right column). Figure S4: DSC thermograms of the neat resins and different composite samples acquired for successive heating and cooling cycles. Figure S5: Strain, coefficient of thermal expansion (*α*), storage modulus (*E*′) and loss tangent (Tan *δ*) in an SLA printed neat PEGDA-250 sample as a function of the temperature. Heating (red) and cooling (blue) refer to the second thermal cycle. Figure S6: Strain, coefficient of thermal expansion (*α*), storage modulus (*E*′) and loss tangent (Tan *δ*) in an SLA printed **1**@DS3000_15 composite sample as a function of the temperature. Heating (red) and cooling (blue) refer to the second thermal cycle. Figure S7: Strain, coefficient of thermal expansion (*α*), storage modulus (*E*′) and loss tangent (Tan *δ*) in an SLA printed **1**@DS3000_20 composite sample as a function of the temperature. Heating (red) and cooling (blue) refer to the second thermal cycle. Figure S8: Strain, coefficient of thermal expansion (*α*), storage modulus (*E*′) and loss tangent (Tan *δ*) in an SLA printed **1**@DS3000_35 composite sample as a function of the temperature. Heating (red) and cooling (blue) refer to the second thermal cycle. Figure S9: Strain, coefficient of thermal expansion (*α*), storage modulus (*E*′) and loss tangent (Tan *δ*) in an SLA printed **1**@PEGDA_30 composite sample as a function of the temperature. Heating (red) and cooling (blue) refer to the second thermal cycle. Figure S10: Strain, coefficient of thermal expansion (*α*), storage modulus (*E*′) and loss tangent (Tan *δ*) in an SLA printed **1**@PEGDA_38 composite sample as a function of the temperature. Heating (red) and cooling (blue) refer to the second thermal cycle. Figure S11: Strain, coefficient of thermal expansion (*α*), storage modulus (*E*′) and loss tangent (Tan *δ*) in an SLA printed **2**@PEGDA_30 composite sample as a function of the temperature. Heating (red) and cooling (blue) refer to the second thermal cycle.
