# Supplementary material for: Stereolithography 3D Printing of Stimuli-Responsive Spin Crossover@Polymer Nanocomposites with Optimized Actuating Properties
Source: Nanomaterials (Basel). 2024 Jul 24;14(15):1243. doi: 10.3390/nano14151243 (PMC11313888; doi:10.3390/nano14151243)
Supplement: Supplementary file 1 [file nanomaterials-14-01243-s001.zip › nanomaterials-3072151-supplementary.pdf]

ELECTRONIC SUPPLEMENTARY INFORMATION

Stereolithography 3D Printing of Stimuli-Responsive Spin Crossover@Polymer Nanocomposites with Optimized Actuating Properties

Onkar Kulkarni[ab], Alejandro Enriquez-Cabrera[a], Xinyu Yang[a], Julie Foncy[b], Liviu Nicu[b], Gábor Molnár*[a], Lionel Salmon*[a]

[a] *LCC, CNRS & University of Toulouse, 205 route de Narbonne, 31077 Toulouse, France*

[b] *LAAS, CNRS & University of Toulouse, 7 avenue du Colonel Roche, 31400 Toulouse, France*

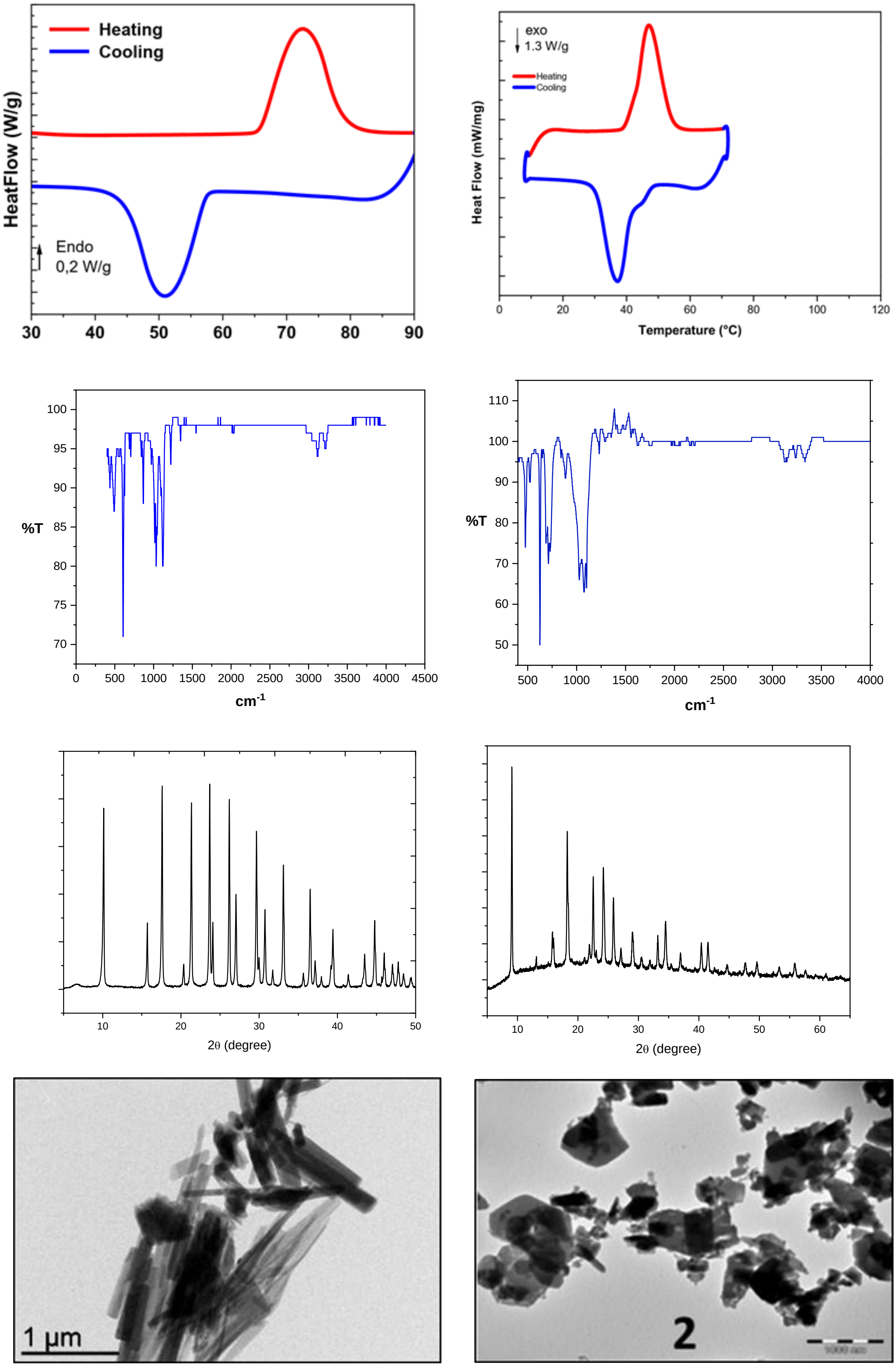

Figure S1. DSC thermograms, FTIR spectra, powder XRD patterns (Cu K-alpha) and representative TEM images for the powder samples of $[Fe(NH_2trz)_3]SO_4$ (left panel) and $[Fe(NH_2trz)_3](BF_4)(SiF_6)_{0.5}$ (right panel).

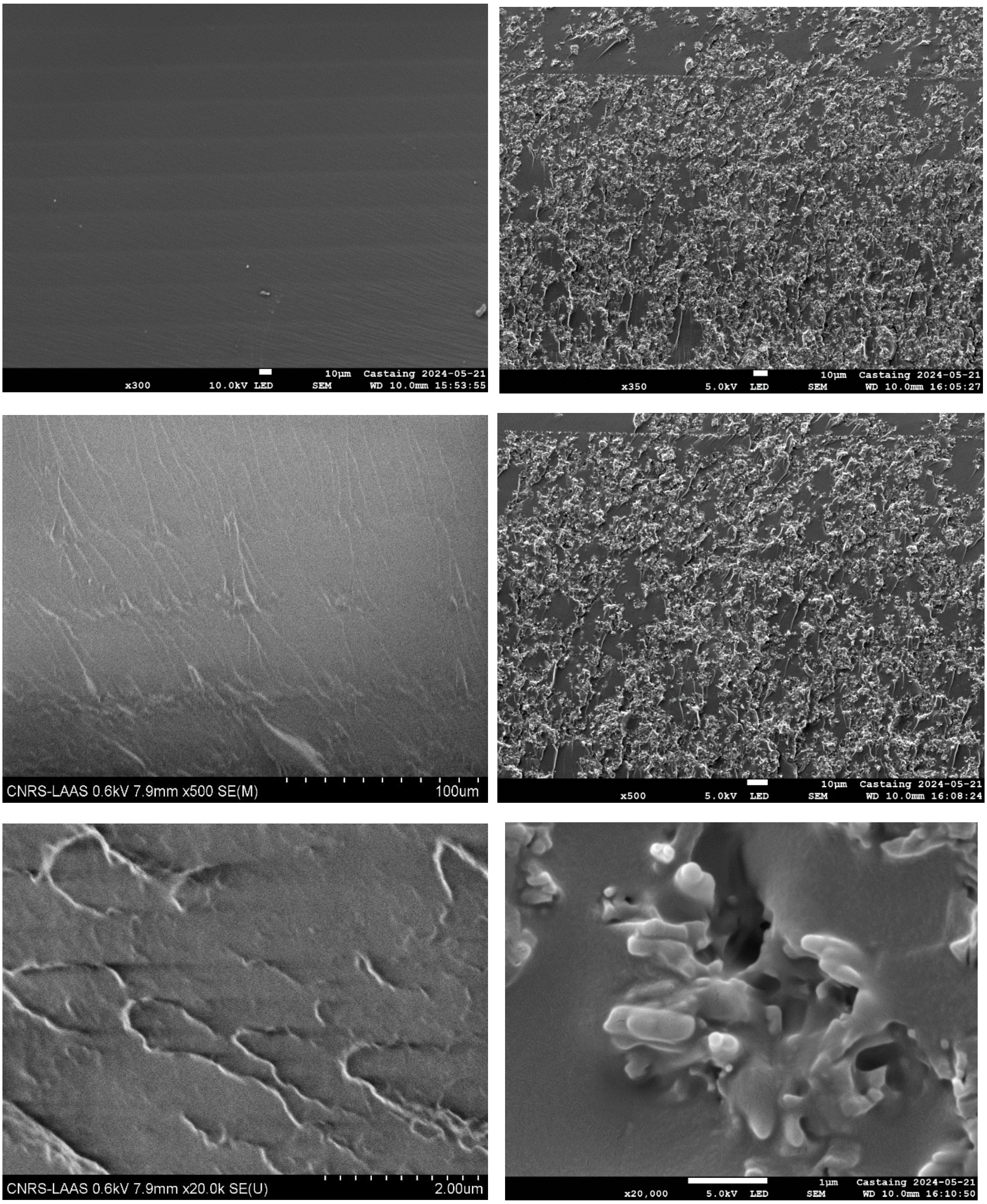

Neat PEGDA **1**@PEGDA (30 wt.%)

Figure S2. Representative SEM images of the neat SLA printed PEGDA film (left column) and the **1**@PEGDA (30 wt.%) composite sample (right column).

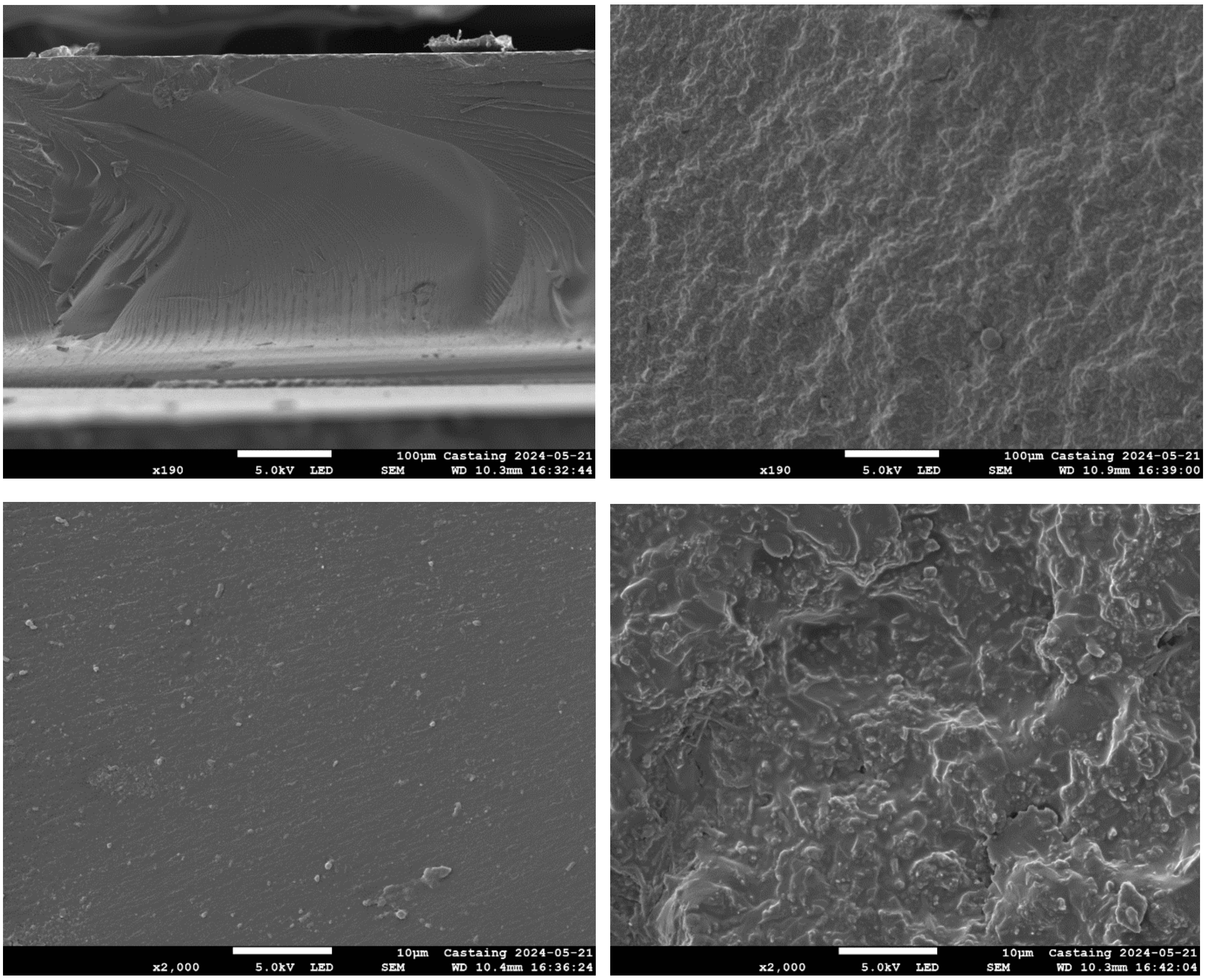

Neat DS3000 **1**@DS3000 (30 wt.%)

Figure S3. Representative SEM images of the neat SLA printed DS3000 film (left column) and the **1**@DS3000 (30 wt.%) composite sample (right column).

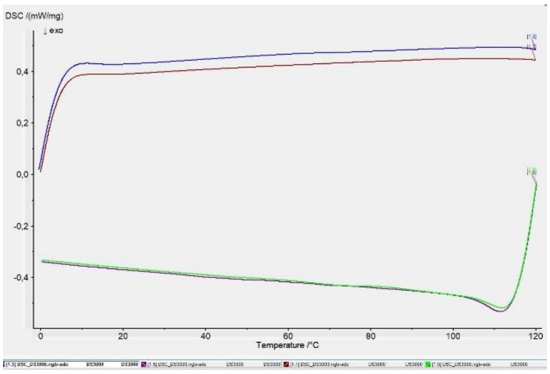

DS3000

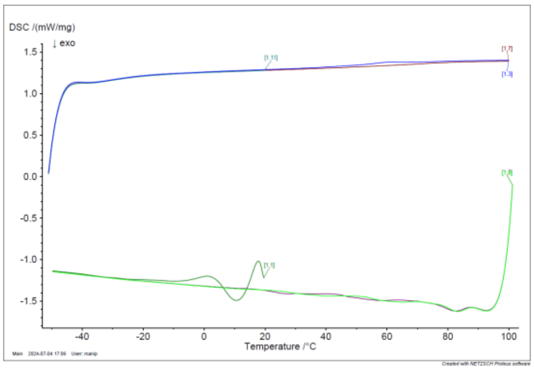

PEGDA-250

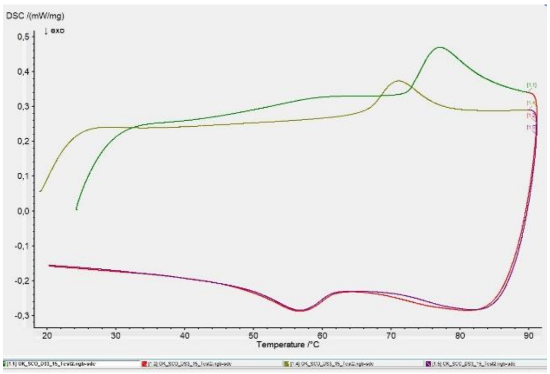

1@DS3000_15

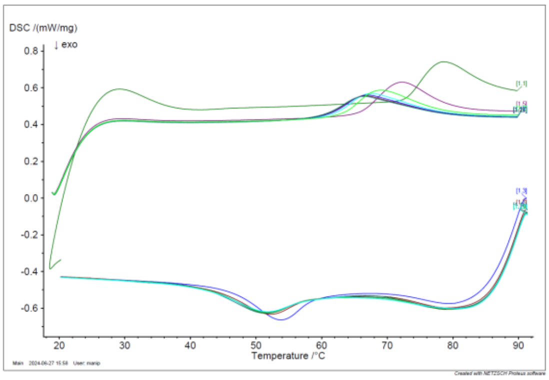

1@DS3000_20

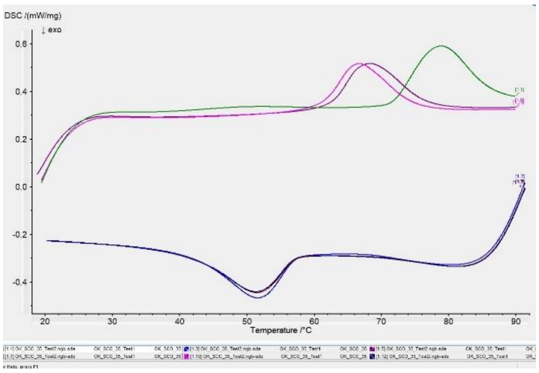

1@DS3000_35

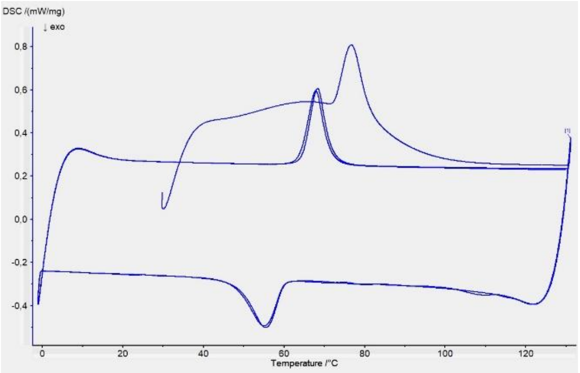

1@PEGDA_30

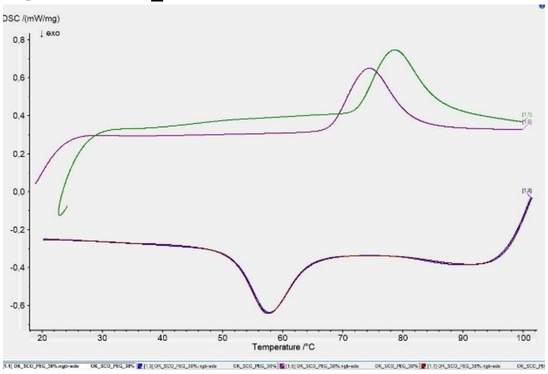

1@PEGDA_38

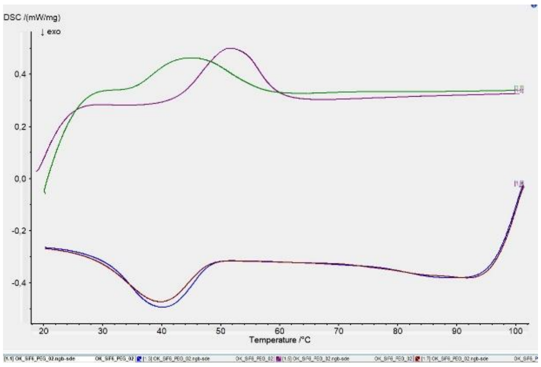

2@PEGDA_30

Figure S4. DSC thermograms of the neat resins and different composite samples acquired for successive heating and cooling cycles.

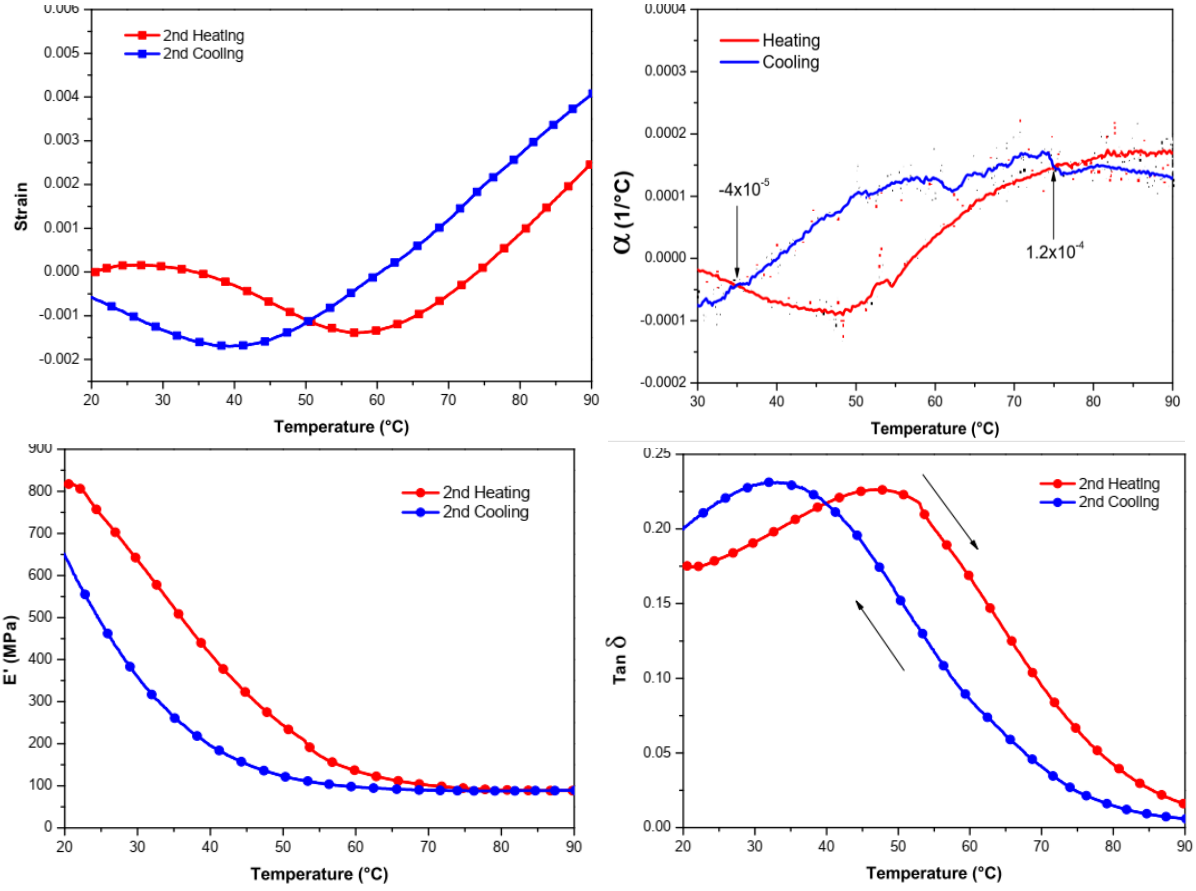

Figure S5. Strain, coefficient of thermal expansion (α), storage modulus (E') and loss tangent (Tan δ) in an SLA printed neat PEGDA-250 sample as a function of the temperature. Heating (red) and cooling (blue) refer to the second thermal cycle.

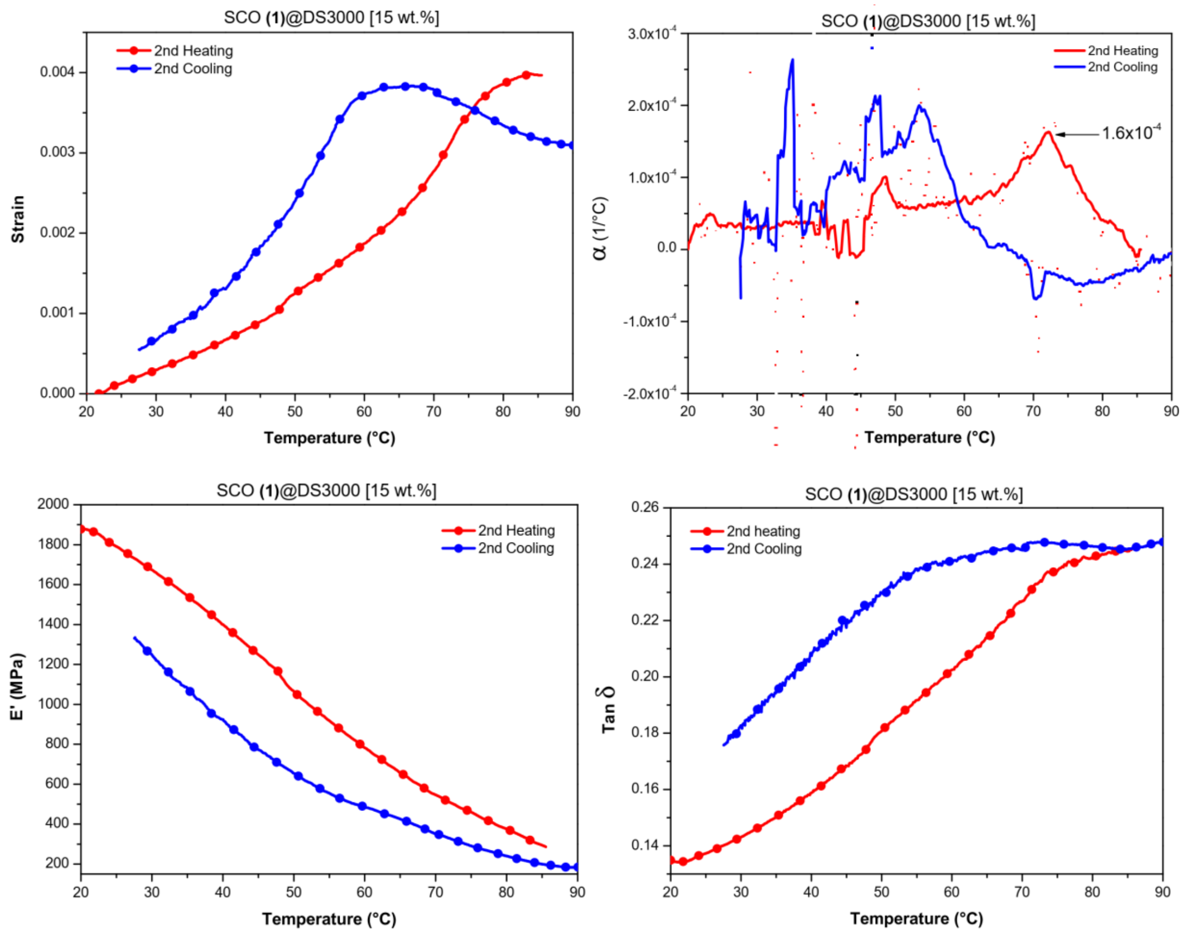

Figure S6. Strain, coefficient of thermal expansion (α), storage modulus (E') and loss tangent (Tan δ) in an SLA printed **1**@DS3000_15 composite sample as a function of the temperature. Heating (red) and cooling (blue) refer to the second thermal cycle.

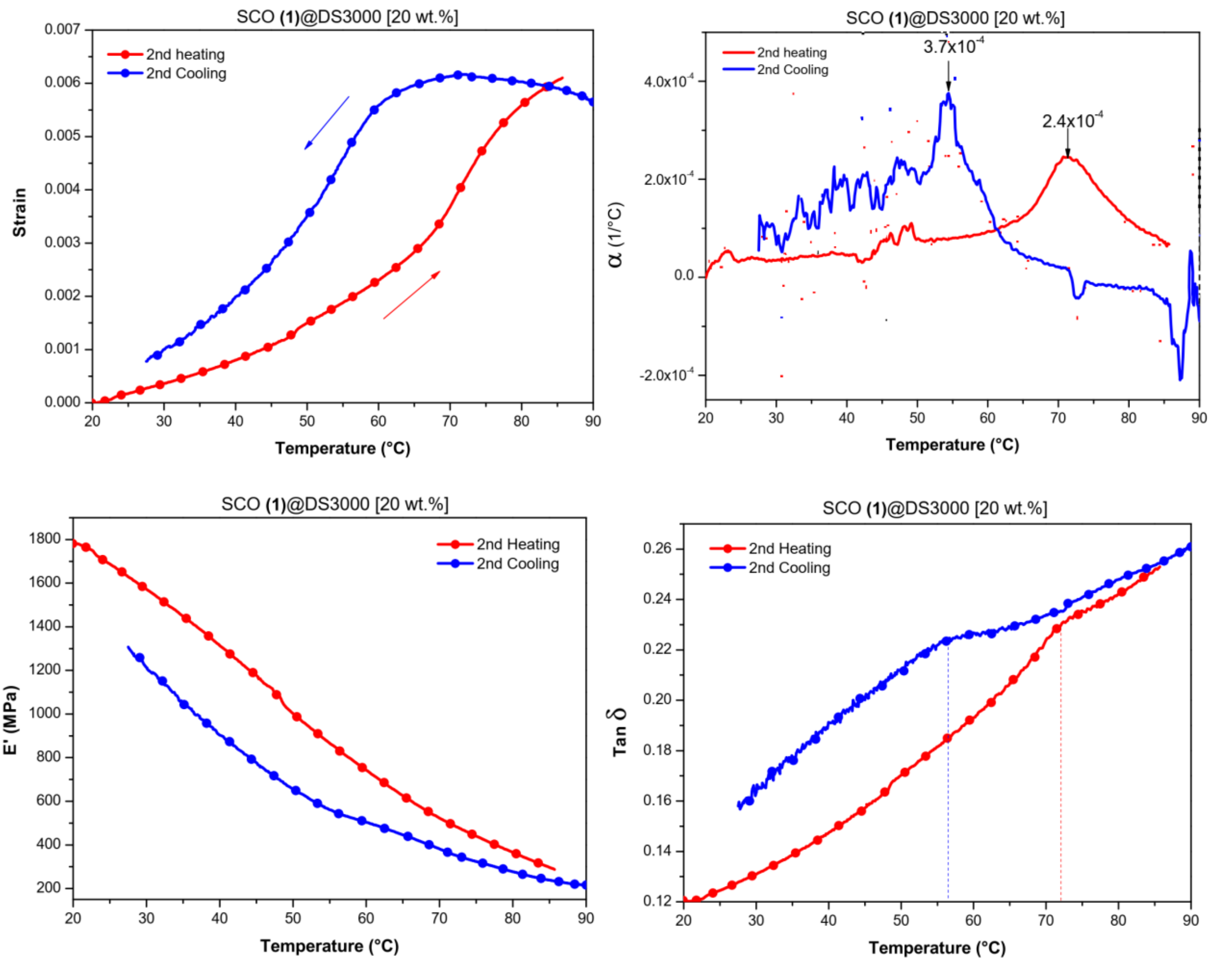

Figure S7. Strain, coefficient of thermal expansion (α), storage modulus (E') and loss tangent (Tan δ) in an SLA printed **1**@DS3000_20 composite sample as a function of the temperature. Heating (red) and cooling (blue) refer to the second thermal cycle.

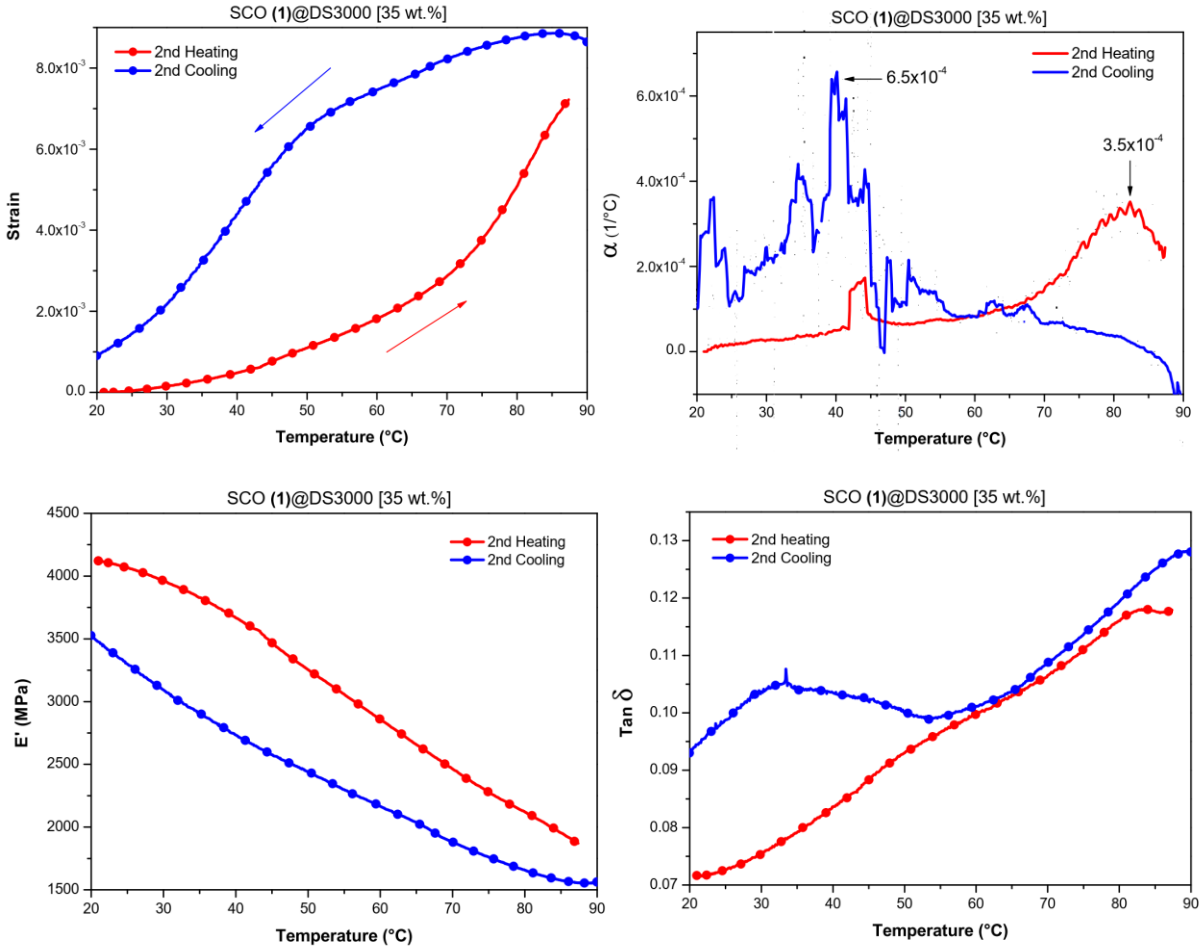

Figure S8. Strain, coefficient of thermal expansion (α), storage modulus (E') and loss tangent (Tan δ) in an SLA printed **1**@DS3000_35 composite sample as a function of the temperature. Heating (red) and cooling (blue) refer to the second thermal cycle.

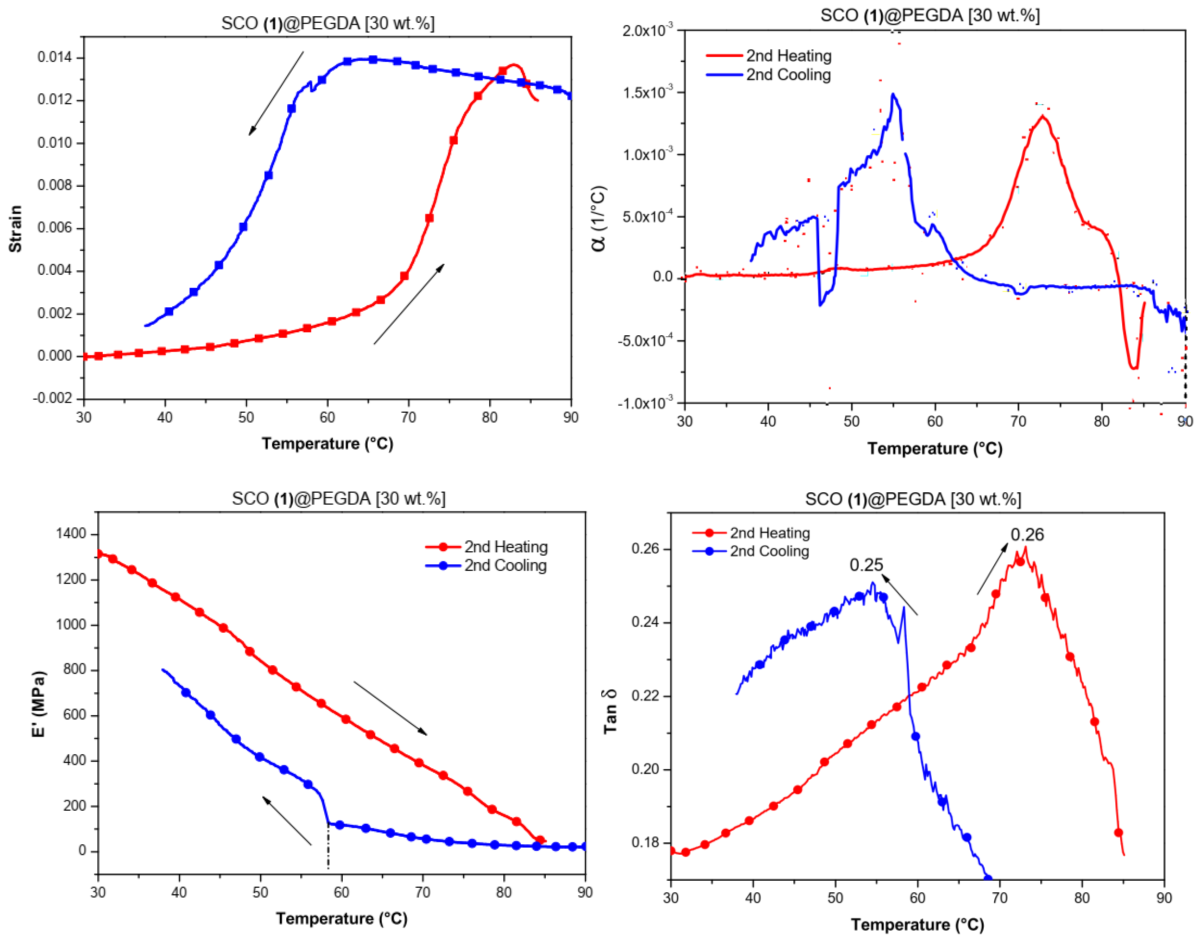

Figure S9. Strain, coefficient of thermal expansion (α), storage modulus (E') and loss tangent (Tan δ) in an SLA printed **1**@PEGDA_30 composite sample as a function of the temperature. Heating (red) and cooling (blue) refer to the second thermal cycle.

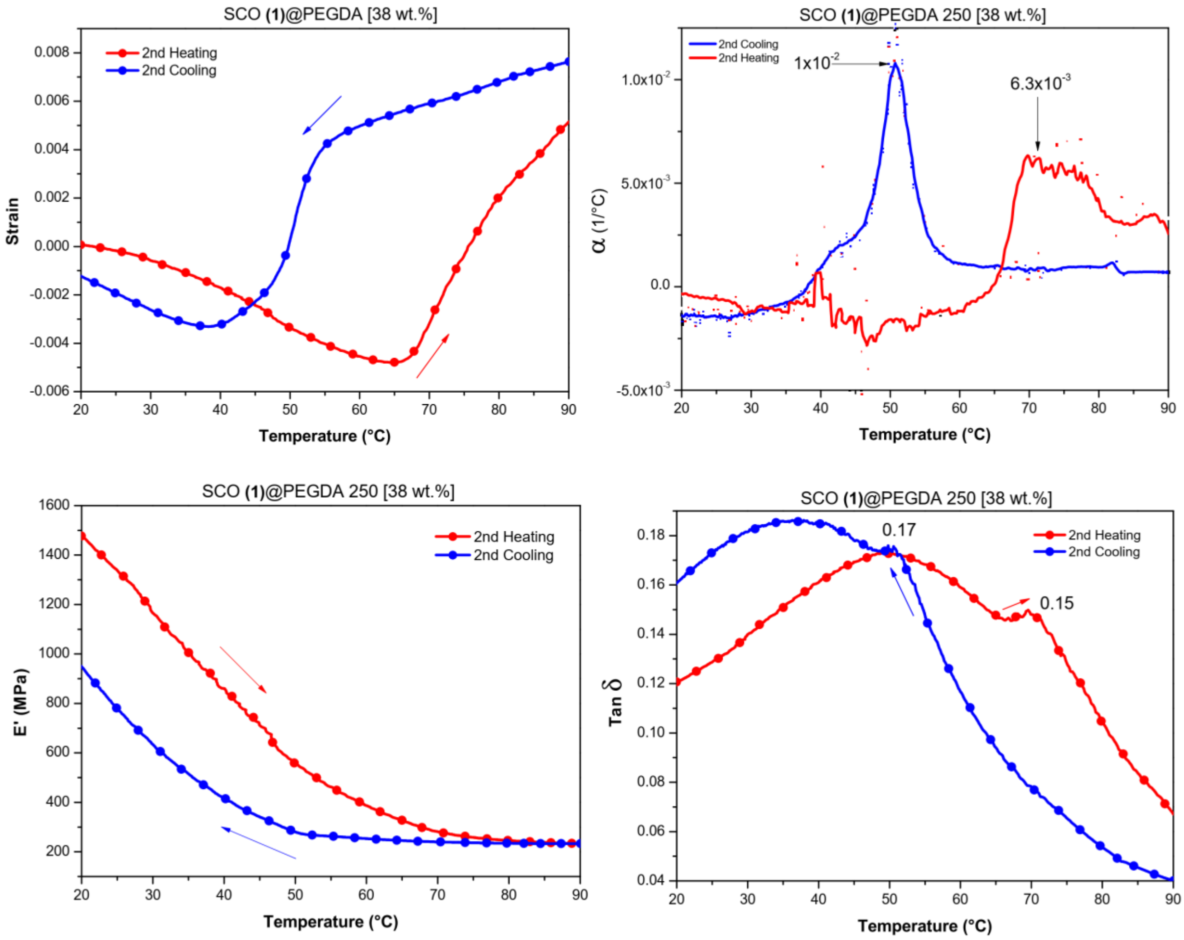

Figure S10. Strain, coefficient of thermal expansion (α), storage modulus (E') and loss tangent (Tan δ) in an SLA printed **1**@PEGDA_38 composite sample as a function of the temperature. Heating (red) and cooling (blue) refer to the second thermal cycle.

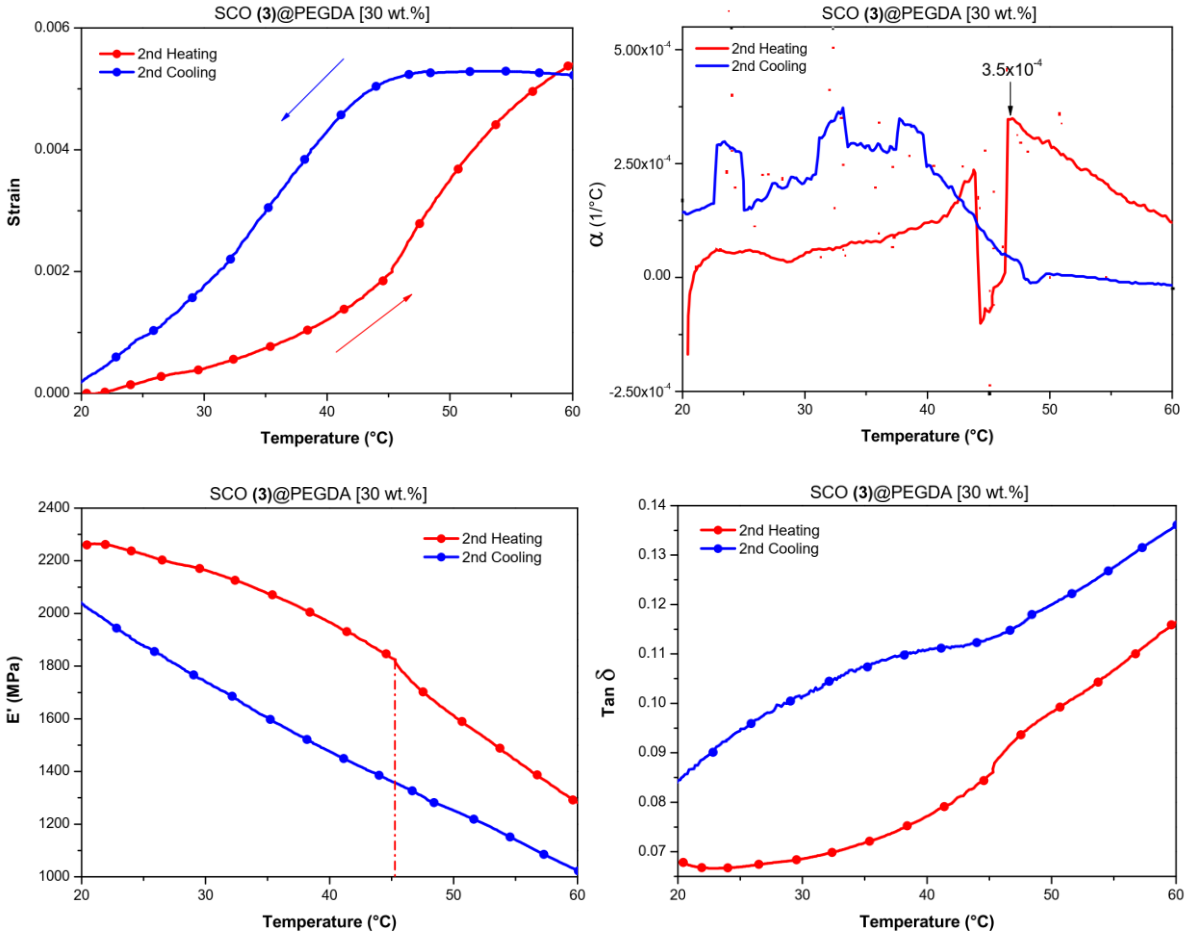

Figure S11. Strain, coefficient of thermal expansion (α), storage modulus (E') and loss tangent (Tan δ) in an SLA printed **2**@PEGDA_30 composite sample as a function of the temperature. Heating (red) and cooling (blue) refer to the second thermal cycle.
